# Comparative Evaluation of the Sealing Ability of Root Canal Sealers: An In Vitro Study Using Dye Penetration and Scanning Electron Microscopic Analysis

**DOI:** 10.7759/cureus.80741

**Published:** 2025-03-17

**Authors:** Nabhika Mittal, Saru Gupta, Saurabh Gupta, Rajinder Bansal, Vishakha Grover, Poonam Bogra, Sanjana Khullar, Seema Gupta

**Affiliations:** 1 Department of Conservative Dentistry and Endodontics, DAV Centenary Dental College, Yamunanagar, IND; 2 Department of Pediatric and Preventive Dentistry, Maharishi Markandeshwar College of Dental Sciences and Research, Ambala, IND; 3 Department of Conservative Dentistry and Endodontics, All India Institute of Medical Sciences, Jammu, IND; 4 Department of Conservative Dentistry and Endodontics, Guru Nanak Dev Dental College and Research Institute, Sunam, IND; 5 Department of Periodontology, Dr. Harvash Singh Judge Institute of Dental Sciences and Hospital, Chandigarh, IND; 6 Department of Orthodontics, Kothiwal Dental College and Research Centre, Moradabad, IND

**Keywords:** cold lateral compaction, methylene blue dye, microleakage, root canal sealer, scanning electron microscopy

## Abstract

Introduction: Achieving an impervious seal in root canal treatment is crucial for preventing bacterial infiltration and ensuring long-term success. Various root canal sealers are available, each with different compositions and sealing abilities. This in vitro study aimed to compare the sealing abilities of four different root canal sealers using dye penetration and scanning electron microscopy (SEM).

Methods: Sixty-two extracted single-rooted human teeth were prepared using a standardized rotary instrumentation technique. One tooth served as the positive control, and one tooth served as the negative control. The remaining 60 samples were randomly divided into four experimental groups. In Group 1 (n = 15), gutta-percha (Dentsply Maillefer, Ballaigues, Switzerland) was used in conjunction with Tubli-Seal (SybronEndo, Orange, California). In Group 2 (n = 15), gutta-percha was used in conjunction with AH Plus (Dentsply Maillefer). In Group 3 (n = 15), gutta-percha was used in conjunction with RealSeal SE (SybronEndo). In Group 4 (n = 15), gutta-percha was used in conjunction with SmartPaste Bio (Smartseal DRFP Ltd., Stamford, UK). The samples were obturated using the cold lateral condensation method. After obturation, 10 teeth in each group were evaluated for microleakage using the dye penetration method, and five teeth from each group were examined under SEM to analyze the adaptation of the sealers to the dentinal walls. The data were statistically analyzed.

Results: The mean dye penetration values were significantly different between groups (P < 0.05). Group 1 showed the highest mean dye infiltration (9.30 ± 1.75 mm), followed by Group 2 (6.80 ± 1.21 mm), Group 3 (4.70 ± 0.89 mm), and the least in Group 4 (2.15 ± 1.16 mm). SEM analysis further confirmed that Group 1 had the highest mean microgap (18.44 ± 4.98 µm), followed by Group 2 (12.90 ± 2.81 µm), Group 3 (9.41 ± 3.51 µm), and the least in Group 4 (2.65 ± 2.07 µm).

Conclusion: The findings suggest that the sealing ability of root canal sealers varies significantly, with SmartPaste Bio demonstrating the best performance in terms of microleakage and adaptation to the dentinal walls. Clinicians should consider the choice of sealer as a critical factor in achieving an optimal apical seal. Further in vivo studies are required to validate these results and determine their clinical implications.

## Introduction

Endodontic treatment aims to treat pulpal infections and prevent reinfections by achieving an impervious seal in the root canal system. An effective root canal sealer (RCS) plays a crucial role in enhancing the sealing ability of obturation materials, preventing microleakage, and ensuring long-term success [1]. Various sealers are available on the market, with different compositions, physical properties, and sealing effectiveness. Evaluating the sealing ability of these sealers is essential to determine their efficacy in preventing bacterial penetration and apical leakage, which are the primary causes of endodontic failure [2].

Microleakage is a critical factor that influences the outcome of root canal therapy. It refers to the penetration of fluids, microorganisms, and their by-products along the interface between the root canal filling material and dentinal walls [3]. Microleakage occurring at the junction of the root canal filling and canal walls can negatively influence treatment outcomes. Apical leakage is a major contributor to endodontic failure [4]. Gutta-percha is regarded as an impermeable core substance; consequently, leakage in an obturated root canal is anticipated to occur at the interfaces between the sealer and dentin, the sealer and gutta-percha, or through defects within the sealer [5]. Therefore, gutta-percha should be used along with a biocompatible sealer to prevent microleakage. Apical sealing is crucial for inhibiting the migration of bacteria and their associated endotoxins [3].

Various methods have been used to assess microleakage, including dye penetration, bacterial leakage, fluid filtration, scanning electron microscopy (SEM), and micro-computed tomography (micro-CT). Among these, dye penetration and SEM analysis are widely utilized techniques because of their accessibility and reliability in evaluating the sealing ability of RCS [3]. The dye penetration method is a simple and cost-effective technique for assessing the extent of microleakage. This method involves immersing sectioned root specimens in a dye solution and then analyzing the depth of dye penetration under a stereomicroscope. It provides a visual representation of microleakage and allows for a comparative analysis between different sealers. Zanatta et al. [6] assessed microleakage using micro-CT and the conventional dye penetration method and found that micro-CT led to lower values. On the other hand, SEM analysis provides a detailed ultrastructural evaluation of the interface between the sealer and dentinal walls. SEM allows high-resolution imaging of the adaptation of sealers, thereby revealing gaps, voids, and penetration patterns at the microscopic level. This method is advantageous because it offers a more precise assessment of sealing ability but requires complex sample preparation and expensive equipment [7].

Tubli-Seal is a zinc oxide-eugenol (ZOE)-based sealer known for its antimicrobial properties and ease of manipulation. However, it has limitations in terms of solubility and dimensional stability [8]. AH Plus (Dentsply Sirona, Bensheim, Germany), an epoxy resin-based sealer, is widely regarded for its superior sealing ability, biocompatibility, and resistance to dissolution [8]. RealSeal SE (SybronEndo, Orange, California) is a self-etching resin-based sealer designed to improve adhesion to dentin and reduce microleakage [9]. SmartPaste Bio, a bioactive sealer, has been introduced with the potential to enhance remineralization and improve long-term sealing [10].

These sealers were selected for the study because they represent commonly used and well-established materials in endodontic practice, each with distinct chemical compositions and properties. Tubli-Seal is a zinc oxide-eugenol-based sealer, AH Plus is an epoxy resin-based sealer, RealSeal SE is a self-etching methacrylate resin-based sealer, and SmartPaste Bio is a bioceramic-based sealer. The variety in their formulation allows for a comparative evaluation of different sealer categories. Other available sealers not included in this study include calcium hydroxide-based sealers, glass ionomer-based sealers, silicone-based sealers, and other bioceramic sealers such as EndoSequence BC Sealer and MTA Fillapex.

The purpose of this in vitro study was to compare the sealing abilities of the four RCS using two different methodologies: dye penetration and SEM analysis. By assessing sealing effectiveness, this study aimed to provide insights into the most reliable sealer for achieving an optimal apical seal.

## Materials and methods

Study design

This in vitro study was conducted at the Department of Conservative Dentistry and Endodontics, DAV Centenary Dental College, Yamunanagar, from May 2024 to September 2024. The Institutional Ethical Committee waived ethical approval, as it was an in vitro study using extracted teeth with patient consent. The study strictly adhered to the principles of the Declaration of Helsinki.

Sample size estimation

Sample size estimation was performed using G*Power software version 3.1 (Heinrich-Heine-Universität Düsseldorf, Düsseldorf, Germany) to achieve 80% statistical power with a 5% significance level (alpha error). A minimum effect size of 0.47, derived from a previous study by Steier et al. [11], was used to determine that 60 teeth would be sufficient for the study. The referenced study assessed the mean marginal gap of 1.33 µm and 3.33 µm in AH Plus and RealSeal SE sealers at the dentin-sealer interface, with a pooled standard deviation of 0.45. The present study was conducted on 62 teeth, with one tooth serving as the negative control and one as the positive control to confirm the reliability and accuracy of the experimental setup.

Sample collection

The identities of the donors were kept confidential. Sixty-two non-cariogenic, single-rooted human premolar teeth extracted for orthodontic treatment were procured for the present investigation from patients aged 18-35 years. After the removal of soft tissue and calcified deposits from the root surfaces using an ultrasonic scaler, the roots of all teeth were examined under magnification to confirm the absence of cracks, fissures, and root caries. All teeth underwent radiographic imaging in buccolingual and mesiodistal orientations to identify those exhibiting single canals with Type 1 root morphology. Carious teeth, teeth with cracks, developmental defects, calcified canals, internal or external root resorption, additional canals, or fillings were excluded. The teeth were subsequently preserved in normal saline, which was changed weekly, and used within three months of the study.

Methodology

All specimens were marked on the buccal aspect of their root surfaces by creating a minor groove using a contra-angle micromotor handpiece for subsequent identification. Among these, 61 teeth were prepared, whereas one tooth remained unprepared and served as a negative control. The crowns of the 61 teeth were excised at the cementoenamel junction (CEJ) using a diamond disc.

The teeth were decoronated using a diamond disc under water-cooling to standardize the root length to 14 mm. Working length was determined by inserting a #10 K-file (Dentsply Maillefer, Ballaigues, Switzerland) into the canal until it was visible at the apical foramen and subtracting 1 mm. Root canal instrumentation was performed using the ProTaper Universal rotary system (Dentsply Maillefer) up to an #F2 file with an endomotor (X Smart, Dentsply Maillefer) at a speed of 250-350 rpm. Different torques were used for the various files according to the manufacturer’s instructions. The canals were irrigated with 5.25% sodium hypochlorite (NaOCl) (Prevest DenPro Ltd., Jammu, India) after each file change. Final irrigation was performed using 17% ethylenediaminetetraacetic acid (EDTA) (Prevest DenPro Ltd.), followed by distilled water and drying with sterile paper points (Prevest DenPro Ltd.). After each use, the instrument was inspected, and the flutes were cleaned of debris.

Of the 61 prepared specimens, one tooth was left without obturation and served as a positive control, whereas the remaining 60 specimens were randomly allocated into four groups, each comprising 15 specimens, based on the type of sealer used during obturation. Group 1 (n = 15) used gutta-percha (Dentsply Maillefer) in conjunction with Tubli-Seal (SybronEndo). Group 2 (n = 15) used gutta-percha in conjunction with AH Plus (Dentsply Maillefer). Group 3 (n = 15) used gutta-percha in conjunction with RealSeal SE (SybronEndo). Group 4 (n = 15) used gutta-percha in conjunction with SmartPaste Bio (Smartseal DRFP Ltd., Stamford, UK). All canals were obturated using the cold lateral condensation technique. This technique is widely used due to its simplicity, cost-effectiveness, and ability to achieve a dense and well-adapted filling with good length control. It allows for effective compaction of gutta-percha against canal walls, minimizing voids and ensuring a satisfactory seal in a variety of canal shapes. The process involved placing tapered #F2 gutta-percha cones into the canal and condensing them under pressure against the canal walls using a finger spreader (Mani Inc., Tochigi, Japan). A tug-back test was used to check the fit of the cone.

Sealers were prepared according to the manufacturer’s guidelines. Tubli-Seal and AH Plus were presented as dual-paste systems comprising a base and a catalyst, which were dispensed in equal lengths on a mixing pad and mixed until a creamy consistency was achieved. The setting duration for Tubli-Seal was 20 minutes on a glass slab and 5 minutes within a root canal, whereas AH Plus had a working time of 4 hours and a setting time of 8 hours. RealSeal SE was available in the form of a dual-syringe apparatus containing a sealing agent and was equipped with an automatic tip that facilitated the application of the sealer onto a mixing pad. The material underwent polymerization upon exposure to light-emitting diode (LED) light with a power output of approximately 1000-1200 mW/cm². SmartPaste Bio was provided as a single-paste formulation and was dispensed onto a mixing pad with a setting time of 24 hours. The samples were stored at 37°C in 100% humidity for seven days to allow complete sealer setting.

The root canal was filled with sealing material using a lentulospiral instrument (Mani Inc., Tochigi, Japan). The sealing agent was introduced into the canal through gradual rotation of the lentulospiral. A small quantity of sealing material was applied to the spiral before insertion into the root canal. During retraction of the spiral from the canal, it was gently pressed against the canal wall. A pre-measured master cone coated with a sealing material was positioned within the canal. The gutta-percha accessory cones were placed into the space formed by the spreader within the canal. This procedure was repeated until the spreader could not advance beyond the cervical third of the canal.

**Figure 1:**
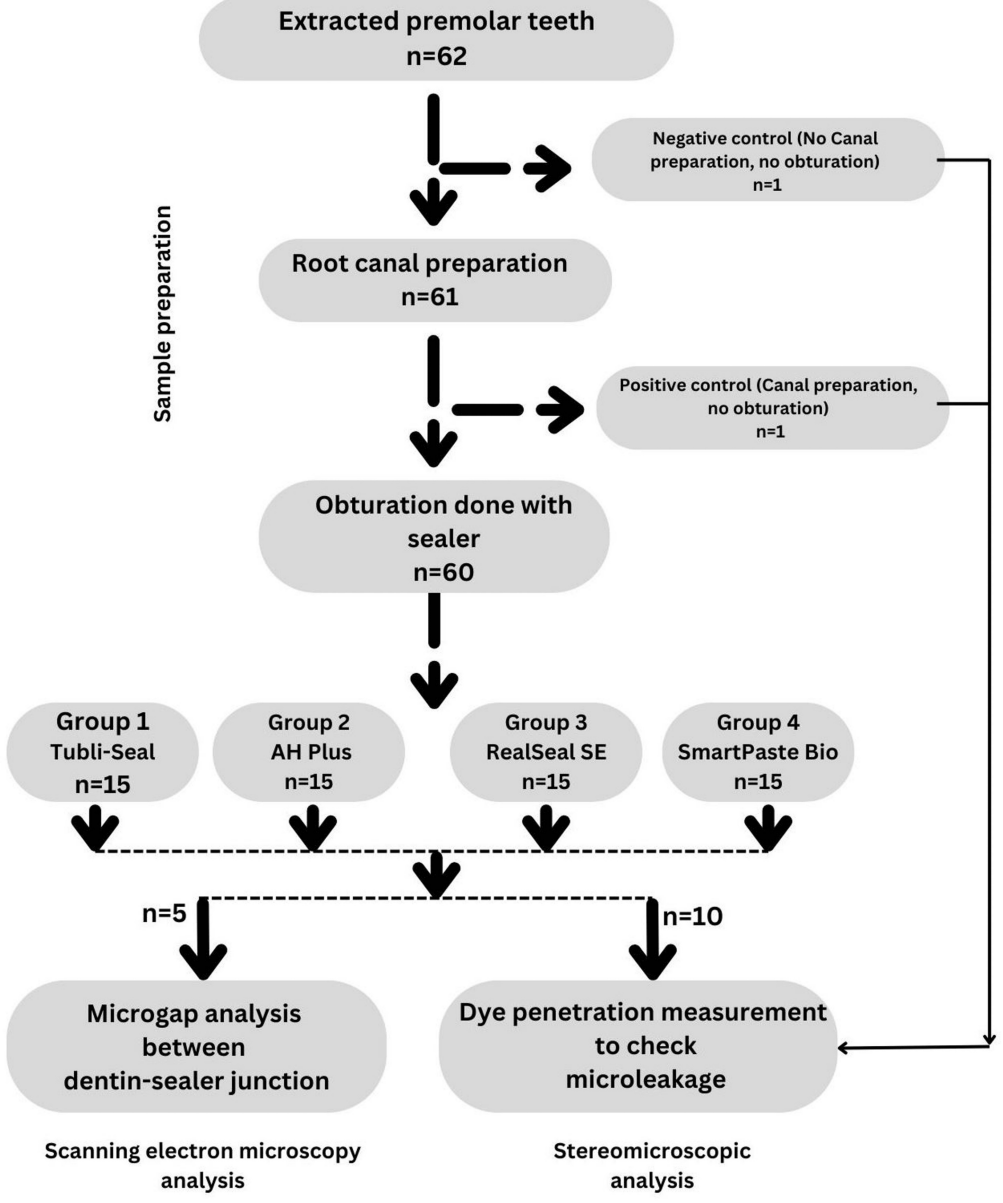
Study flowchart.

After insertion of the cones, they were meticulously sealed at the orifice using a heated burnisher, ensuring that an additional 2 mm of gutta-percha was excised from the cervical region of the canal, while the remaining filling material was compacted with the assistance of a plugger (Mani Inc., Tochigi, Japan). Following gutta-percha extraction, a layer of temporary cement, specifically Cavit G (3M, St. Paul, Minnesota), was applied atop the cone to serve as a definitive cap, mitigating the risk of coronal leakage. Intraoral periapical (IOPA) radiographic images were obtained to evaluate the integrity and quality of the obturation process. All specimens were maintained in an incubator at 100% humidity for one week (Figure 1).

**Figure 2:**
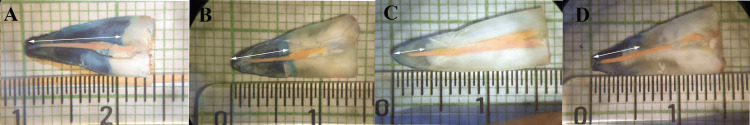
Dye penetration estimated by stereomicroscope at 20x magnification in (A) Group 1, (B) Group 2, (C) Group 3, and (D) Group 4. This image is of the samples from the study.

A microleakage assessment was conducted using two different methodologies to evaluate the sealing ability of the tested RCS: the dye penetration method and SEM analysis. Ten (67%) teeth in each group underwent dye penetration analysis, and five (33%) teeth in each group underwent SEM analysis. For the dye penetration method, the external surfaces of the roots were coated with two layers of nail varnish, leaving the apical 2 mm exposed. The positive control tooth was covered in the same manner, whereas the entire tooth from the negative control was completely covered with nail varnish. The specimens were then immersed in a 2% methylene blue dye solution for seven days at 37°C. After the immersion period, the roots were sectioned longitudinally using a diamond disc, and the depth of dye penetration was measured under a stereomicroscope at 20× magnification (Biotron Healthcare, Mumbai, India). The degree of microleakage was assessed in millimeters by measuring the infiltration of dye from the root apex towards the coronal portion (Figure 2).

**Figure 3:**
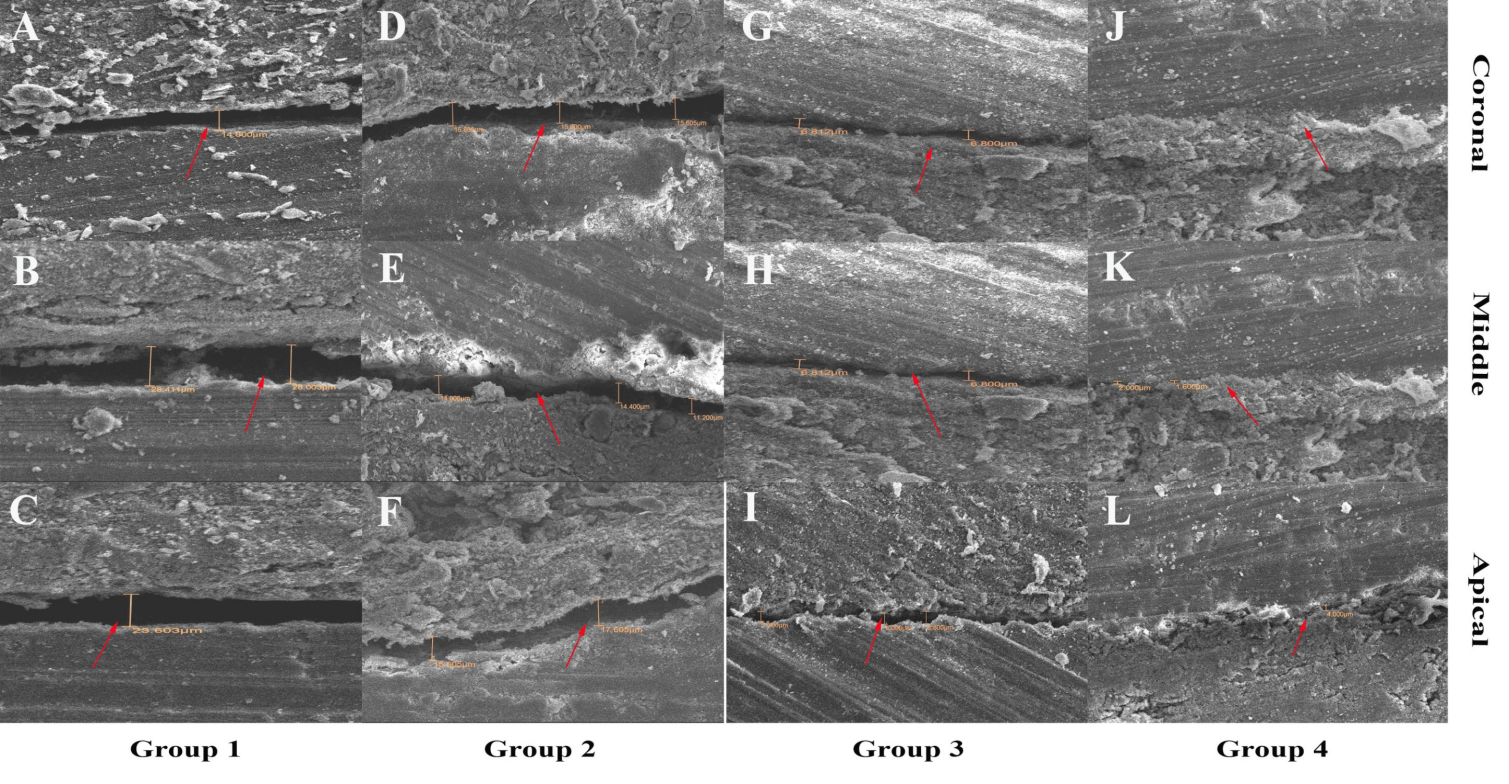
Microgap estimation using a scanning electron microscope at 1000x magnification in Group 1 at (A) coronal, (B) middle, (C) Apical; in Group 2 at (D) coronal, (E) middle, (F) apical; in Group 3 at (G) coronal, (H) middle, (I) apical; in Group 4 at (J) coronal, (K) middle, (L) apical. This figure is of the samples from the study.

For the SEM analysis, the specimens were split longitudinally, and half of each root was prepared for microscopic examination. The samples were dehydrated using increasing ethanol concentrations and subsequently sputter-coated with gold to enhance conductivity (JFC 1600, Jeol Ltd., Tokyo, Japan). The interface between the sealer and dentinal walls was assessed using SEM at a magnification of 500× (JSM-6510LV, Jeol Ltd.) in three regions of the root: the coronal third, middle third, and apical third, to observe adaptation, voids, and gaps in the sealer-dentin interface (Figure 3).

Statistical analysis

Data analysis was performed using IBM SPSS Statistics for Windows (version 23.0; Armonk, New York). The Kolmogorov-Smirnov test was used to assess data normality, confirming a normal distribution. Consequently, parametric tests were used, with one-way analysis of variance (ANOVA) employed to compare the mean microgap measurements obtained using SEM at the cervical, middle, and apical regions, as well as dye infiltration among the groups. To identify significant differences, post-hoc pairwise comparisons were conducted using the Tukey test. A P-value of less than 0.05 was considered significant.

## Results

A significant difference was observed in the mean dye infiltration values between all groups (P = 0.001). Group 1 showed the highest mean dye infiltration (9.30 ± 1.75 mm), followed by Group 2 (6.80 ± 1.21 mm), Group 3 (4.70 ± 0.89 mm), and the least in Group 4 (2.15 ± 1.16 mm). This suggests that sealing efficacy improved across the groups, with Group 4 demonstrating the most effective barrier against dye penetration (Table 1).

**Table 1:** Comparison of mean dye infiltration (in mm) at dentin-sealer interface between groups by one way analysis of variance (ANOVA) test. The data are presented in the form of mean and standard deviation (SD). *P-value <0.05 is considered significant. CI: confidence interval.

Group	N (%)	Mean	SD	95% CI for mean	F-value	P-value
Group 1	10 (25)	9.30	1.75	8.05-10.55	55.69	0.001*
Group 2	10 (25)	6.80	1.21	5.94-7.66		
Group 3	10 (25)	4.70	0.89	4.06-5.34		
Group 4	10 (25)	2.15	1.16	1.32-2.98		

Post-hoc pairwise comparisons revealed statistically significant differences in dye infiltration between the groups (P = 0.001). Group 1 showed significantly higher dye infiltration than Groups 2, 3, and 4, with the largest difference observed between Groups 1 and 4. Similarly, Group 2 exhibited significantly greater infiltration than Groups 3 and 4, whereas Group 3 demonstrated a statistically significant difference compared to Group 4. These findings indicate a progressive reduction in dye infiltration across the groups, suggesting that sealing ability improves with the materials or techniques used in Groups 3 and 4. The significant differences highlight the effectiveness of Group 4 in minimizing dye penetration, reinforcing its superior sealing performance (Table 2).

**Table 2:** Post-hoc Tukey pairwise comparison for dye infiltration (in mm). *P-value <0.05 is considered significant.

Pairwise comparison	Mean difference	Standard error	t-value	P-value
Group 1-Group 2	2.50	0.577	4.34	0.001*
Group 1-Group 3	4.60	0.577	7.98	0.001*
Group 1-Group 4	7.15	0.577	12.40	0.001*
Group 2-Group 3	2.10	0.577	3.64	0.004*
Group 2-Group 4	4.65	0.577	8.07	0.001*
Group 3-Group 4	2.55	0.577	4.42	0.001*

A significant difference was observed in the mean microgap at the dentin-sealer interface between the groups (P = 0.001). Group 1 showed the highest mean microgap (18.44 ± 4.98 µm), followed by Group 2 (12.90 ± 2.81 µm), Group 3 (9.41 ± 3.51 µm), and the least in Group 4 (2.65 ± 2.07 µm). This suggests that sealing effectiveness improved progressively, with Group 4 demonstrating the most efficient adaptation at the dentin-sealer interface (Table 3).

**Table 3:** Comparison of mean microgap (in µm) at dentin-sealer interface between groups by one way analysis of variance (ANOVA) test. The data are presented in the form of mean and standard deviation (SD). *P-value <0.05 is considered significant. CI: confidence interval.

Group	N (%)	Mean	SD	95% CI for mean	F-value	P-value
Group 1	5 (25	18.44	4.98	15.68-21.20	53.17	0.001*
Group 2	5 (25)	12.90	2.81	11.34-14.46		
Group 3	5 (25)	9.41	3.51	7.46-11.35		
Group 4	5 (25)	2.65	2.07	1.51-3.80		

Post-hoc pairwise comparisons demonstrated significant differences in microgap measurements between most groups (P = 0.001), except for the comparison between Groups 2 and 3 (P = 0.051), which was not statistically significant. Group 1 exhibited significantly higher microgap values than Groups 2, 3, and 4, with the largest difference observed between Groups 1 and 4. Similarly, Group 2 showed a significantly greater microgap than Group 4, whereas Group 3 differed significantly from Group 4. These findings indicate a progressive reduction in microgap size across the groups, with Group 4 demonstrating the most effective adaptation at the dentin-sealer interface (Table 4).

**Table 4:** Post-hoc Tukey pairwise comparison between the groups for microgap analysis (in µm) at dentin-sealer interface. *P-value <0.05 is considered significant.

Pairwise comparison	Mean difference	Standard error	t-value	P-value
Group 1-Group 2	5.54	1.282	4.32	0.001*
Group 1-Group 3	9.03	1.282	7.05	0.001*
Group 1-Group 4	15.79	1.282	12.31	0.001*
Group 2-Group 3	3.49	1.282	2.72	0.051
Group 2-Group 4	10.25	1.282	7.99	0.001*
Group 3-Group 4	6.75	1.282	5.27	0.001*

Intergroup comparisons of microgap measurements at the dentin-sealer interface across different locations (cervical, middle, and apical thirds) revealed significant differences (P = 0.001). Group 1 consistently exhibited the highest microgap values at all three locations, followed by Groups 2 and 3, whereas Group 4 demonstrated the lowest values. The microgap was highest at the apical third for all groups, indicating increased difficulty in achieving an optimal seal in this region. The significant reduction in microgap measurements in Group 4 across all locations suggests superior sealing ability compared with the other groups (Figure 3).

**Figure 4:**
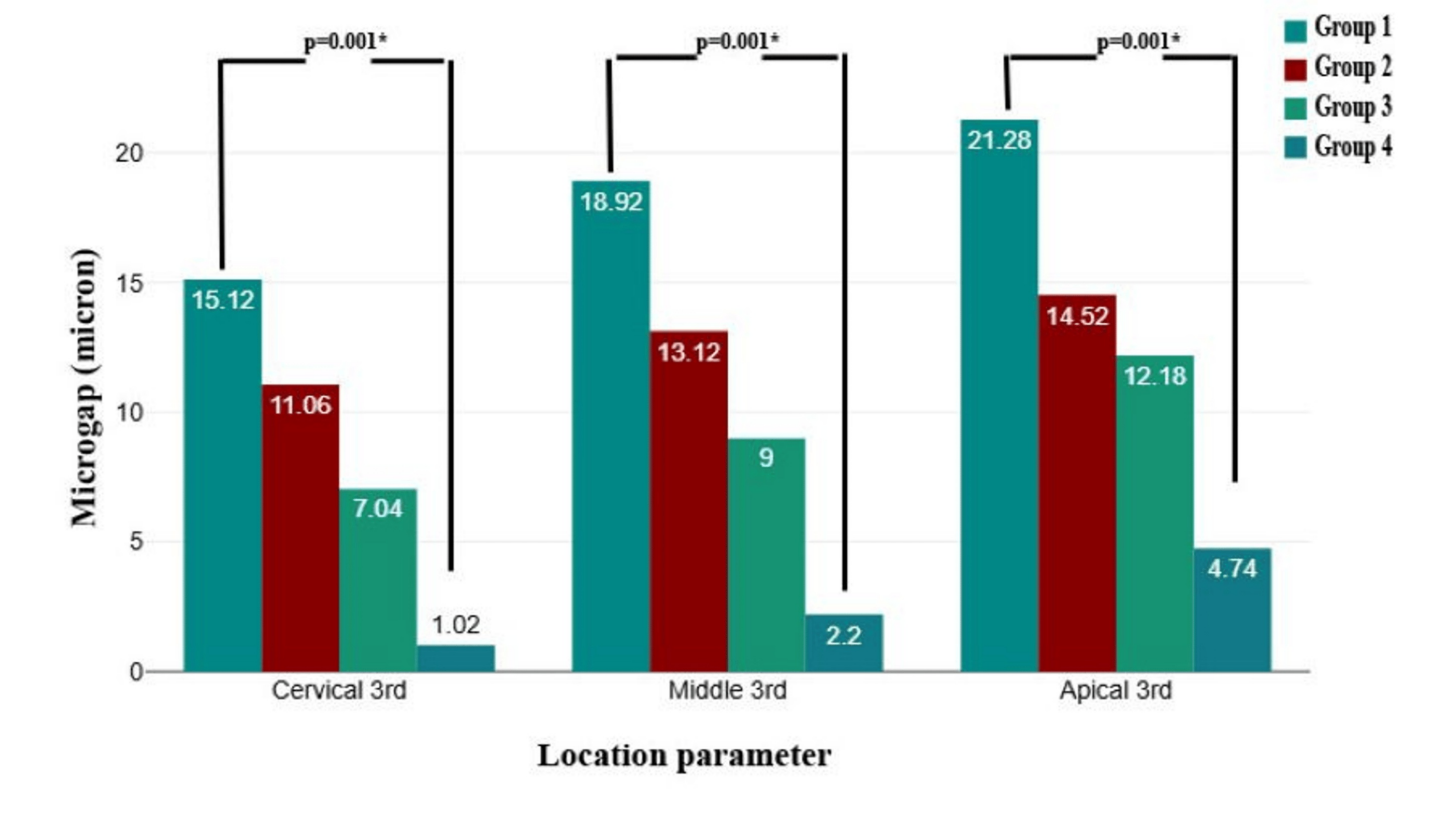
Intergroup comparison of microgap measurements at the dentin-sealer interface across different locations by one way analysis of variance (ANOVA) test. *P-value <0.05 is considered significant. This figure is based on the data from this study.

Intragroup comparison of microgap measurements at the dentin-sealer interface across different locations (cervical, middle, and apical thirds) revealed varying levels of statistical significance. Group 4 exhibited a significant difference between the locations (P = 0.004), with the highest microgap observed in the apical third. In contrast, Groups 1, 2, and 3 did not show statistically significant differences among the three regions (P > 0.05), indicating a relatively uniform microgap distribution within these groups. The significant difference observed in Group 4 emphasizes its improved adaptation at different dentin-sealer interfaces, particularly at the cervical and middle thirds, compared with the other groups (Figure 4).

**Figure 5:**
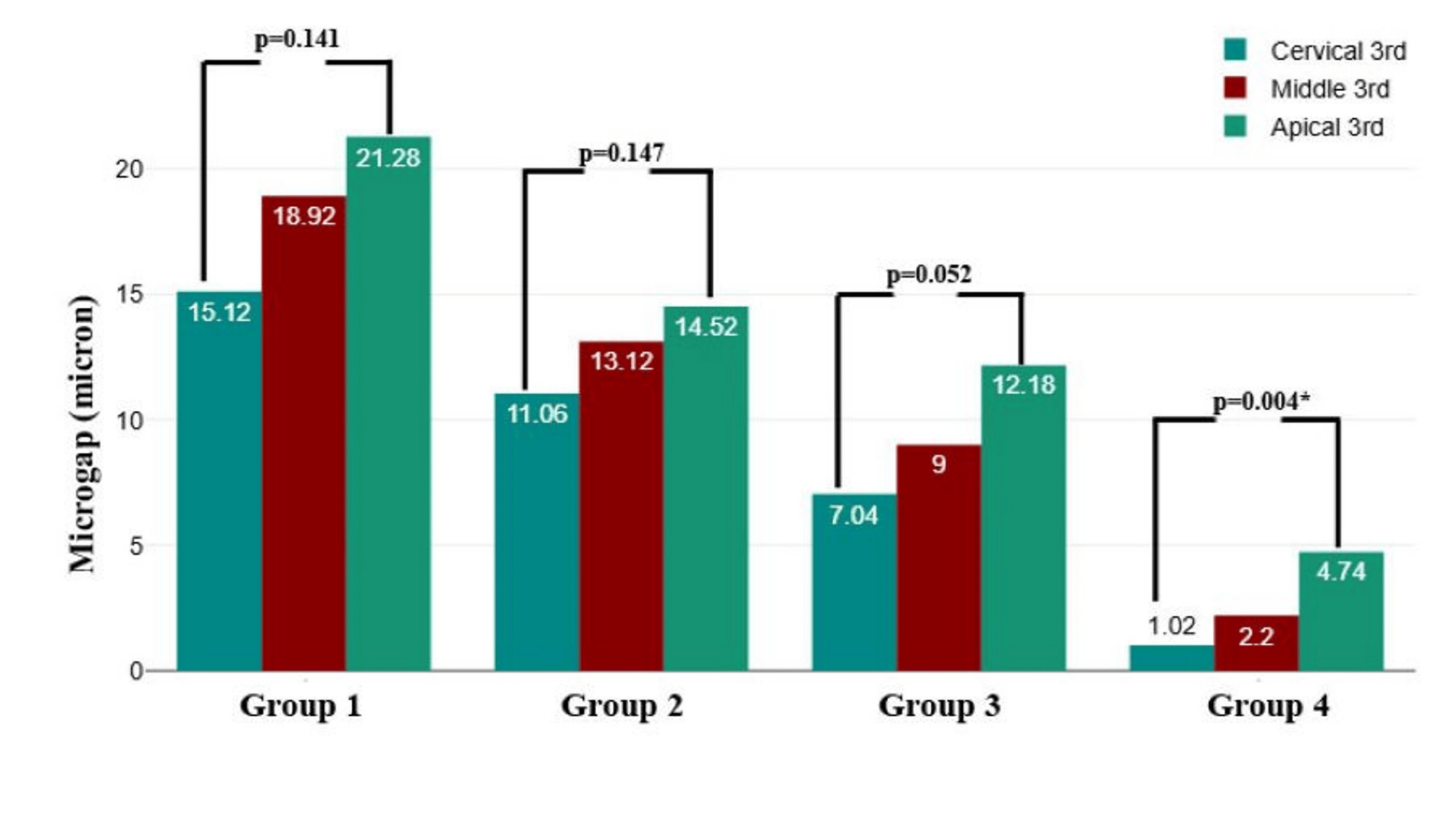
Intragroup comparison of microgap measurements at the dentin-sealer interface across different locations by one way analysis of variance (ANOVA) test. *P-value <0.05 is considered significant. This figure is based on data from this study.

Post-hoc results for intergroup comparisons of microgaps at different locations (cervical, middle, and apical thirds) revealed statistically significant differences in multiple pairwise comparisons. In the cervical third, significant differences were observed between all groups (P < 0.05), indicating variations in microgaps among the different materials or techniques. In the middle third, significant differences were found between Groups 1 and 3, Groups 1 and 4, and Groups 2 and 4 (P < 0.05), whereas other comparisons did not show statistical significance (P > 0.05). Similarly, in the apical third, significant differences were observed in most comparisons, except between Groups 2 and 3 (P = 0.663) (Table 5).

**Table 5:** Post-hoc Tukey test for pairwise comparison of mean microgap (in µm) at cervical, middle, and apical thirds. *P-value <0.05 is considered significant.

Location	Pairwise group	Mean difference	Standard error	P-value
Cervical third	Group 1-Group 2	4.06	1.381	0.043*
	Group 1-Group 3	8.08	1.381	0.001*
	Group 1-Group 4	14.10	1.381	0.001*
	Group 2-Group 3	4.02	1.381	0.045*
	Group 2-Group 4	10.04	1.381	0.001*
	Group 3-Group 4	6.02	1.381	0.002*
Middle third	Group 1-Group 2	5.80	2.341	0.102
	Group 1-Group 3	9.92	2.341	0.003*
	Group 1-Group 4	16.72	2.341	0.001*
	Group 2-Group 3	4.12	2.341	0.327
	Group 2-Group 4	10.92	2.341	0.001*
	Group 3-Group 4	6.80	2.341	0.046*
Apical third	Group 1-Group 2	6.76	2.027	0.020*
	Group 1-Group 3	9.10	2.027	0.002*
	Group 1-Group 4	16.54	2.027	0.001*
	Group 2-Group 3	2.34	2.027	0.663
	Group 2-Group 4	9.78	2.027	0.001*
	Group 3-Group 4	7.44	2.027	0.010*

## Discussion

The present study aimed to evaluate the sealing ability of four different RCS, that is, Tubli-Seal, AH Plus, RealSeal SE, and SmartPaste Bio, using dye penetration and SEM analysis. The findings demonstrated significant differences in microleakage among the groups, with SmartPaste Bio exhibiting the least microleakage and the best adaptation at the dentin-sealer interface.

Dye penetration is a widely accepted method for assessing the sealing ability of RCS. Our results showed that Tubli-Seal exhibited the highest mean dye infiltration, whereas SmartPaste Bio exhibited the lowest. These findings indicate that SmartPaste Bio provided the most effective apical seal, significantly reducing the extent of microleakage compared with the other groups. This phenomenon may be attributed to the composition of Tubli-Seal, which is formulated as a ZOE-based sealing agent. Although ZOE-based sealers are recognized for their antimicrobial characteristics and favorable handling properties, they are characterized by relatively high solubility, which poses a risk to the integrity of the seal over time [12]. These sealers comprise weakly interconnected zinc oxide particles within a zinc eugenolate matrix. When subjected to moisture, hydrolysis occurs, resulting in the release of eugenol and zinc hydroxide [13]. AH Plus performed better than Tubli-Seal because it is an epoxy resin-based sealer with adequate dimensional stability [13]. However, its relatively high viscosity may limit its ability to penetrate the dentinal tubules effectively, potentially explaining why RealSeal SE and SmartPaste Bio exhibited even lower leakage values.

RealSeal SE, a self-etching resin-based sealer, demonstrated superior sealing ability compared with Tubli-Seal and AH Plus. The self-etching property of RealSeal SE may have enhanced its penetration into the dentinal tubules, improving adaptation and bond strength [14]. Babb et al. [15] conducted a study comparing self-etching and non-etching RCS and concluded that RealSeal SE exhibited greater push-out bond strength than other sealers, likely due to its ability to create more intimate contact with dentin.

SmartPaste Bio comprises bioactive glass particles that can induce hydroxyapatite formation upon interaction with bodily fluids. This bioactive characteristic significantly enhances the adaptation of the sealer to dentin by establishing a mineralized barrier, thereby reducing microleakage. Unlike other sealers that rely on mechanical retention, SmartPaste Bio forms a chemical bond with dentin, augmenting its sealing efficacy and minimizing the occurrence of gaps at the dentin-sealer interface. SmartPaste Bio also demonstrates low solubility, allowing it to remain intact and preserve its sealing integrity over an extended period. Additionally, it exhibited minimal shrinkage, preventing the development of microgaps. The formulation of this sealer facilitates effective penetration into the dentinal tubules, reinforcing the sealer-dentin interface and reducing the likelihood of microleakage [16].

SEM analysis provided further insights into the sealing ability of the tested sealers by assessing the microgap at the dentin-sealer interface. The mean microgap values were highest in Tubli-Seal, followed by AH Plus, RealSeal SE, and SmartPaste Bio. These findings correlated well with the dye penetration results, reinforcing the superior sealing ability of SmartPaste Bio.

The large microgap observed in Tubli-Seal may be attributed to its shrinkage upon setting, leading to gap formation at the dentin-sealer interface. Additionally, the lack of chemical bonding between Tubli-Seal and dentin may contribute to its inferior sealing ability [13]. AH Plus exhibited lower microgap values than Tubli-Seal, likely due to its better adhesion and reduced solubility. However, it still demonstrated higher microgap values than RealSeal SE and SmartPaste Bio, suggesting that resin-based and bioactive sealers may offer better adaptation [17,18]. RealSeal SE may have created a stronger bond with dentin, thereby reducing microleakage. However, the presence of gaps and voids in certain areas suggests that further improvements in the formulation are necessary to achieve optimal sealing. SmartPaste Bio exhibited the lowest microgap values across all evaluated regions (cervical, middle, and apical thirds).

Our study further indicated that the microgap was highest in the apical third for all groups, highlighting the continued challenge of achieving an optimal seal in this region [19]. De Almeida et al. [20] found that AH Plus exhibited better sealing ability than ZOE-based sealers. The significant reduction in microgap measurements in SmartPaste Bio across all regions underscores its superior adaptability compared with other sealers.

Strengths of the study

This study utilized a robust methodology incorporating two reliable techniques (dye penetration and SEM analysis) to assess microleakage. The sample size was statistically justified, and strict inclusion criteria ensured uniformity. The study design allowed for a comparative evaluation of multiple sealers under standardized conditions.

Clinical implications of the study

These findings suggest that bioactive sealers, such as SmartPaste Bio, provide superior sealing ability and may improve long-term endodontic success. Clinicians should carefully consider sealer selection, particularly in cases requiring optimal apical sealing. This study underscores the importance of minimizing microleakage to prevent bacterial penetration and enhance the outcomes of root canal therapy.

Limitations of the study

In vitro conditions, including mechanical stress and bacterial dynamics, may not fully replicate the complexity of the oral environment. This study assessed short-term sealing ability without evaluating long-term degradation. Additionally, only the cold lateral condensation technique was used, which may have influenced the outcomes. Future studies should explore other obturation techniques and their clinical performance.

Future recommendations

Further in vivo studies are required to validate these findings under clinical conditions. Long-term evaluations are needed to assess sealer degradation and resistance to bacterial penetration. Investigating additional obturation techniques and incorporating advanced imaging methods, such as micro-CT, could provide deeper insights into sealer adaptation and microleakage prevention.

## Conclusions

The findings of this study revealed statistically significant variations in microleakage and microgap formation across the evaluated groups, with SmartPaste Bio demonstrating minimal microleakage and optimal adaptation at the dentin-sealer interface in all regions. Tubli-Seal exhibited the highest degree of microleakage, followed by AH Plus and RealSeal SE. The enhanced efficacy of SmartPaste Bio may be attributed to its bioactive characteristics, chemical adhesion properties, and reduced solubility. These results indicate that SmartPaste Bio is a promising sealing agent for superior apical sealing, potentially enhancing the long-term outcomes of endodontic interventions.

## References

[1] Chandak M, Patel A, Patel S, Agrawal P, Chandak R, Ikhar A (2024). Clinical utility index for root canal sealers. BMC Oral Health.

[2] Patni PM, Chandak M, Jain P, Patni MJ, Jain S, Mishra P, Jain V (2016). Stereomicroscopic evaluation of sealing ability of four different root canal sealers - an in vitro study. J Clin Diagn Res.

[3] Veríssimo DM, do Vale MS (2006). Methodologies for assessment of apical and coronal leakage of endodontic filling materials: a critical review. J Oral Sci.

[4] Cobankara FK, Orucoglu H, Sengun A, Belli S (2006). The quantitative evaluation of apical sealing of four endodontic sealers. J Endod.

[5] Royer K, Liu XJ, Zhu Q, Malmstrom H, Ren YF (2013). Apical and root canal space sealing abilities of resin and glass ionomer-based root canal obturation systems. Chin J Dent Res.

[6] Ferreira Zanatta R, Wiegand A, Dullin C, Bühler-Borges A, Torres C, Rizk M (2019). Comparison of micro-CT and conventional dye penetration for microleakage assessment after different aging conditions. Int J Adhes Adhesives.

[7] Mohammadian F, Farahanimastary F, Dibaji F, Kharazifard MJ (2017). Scanning electron microscopic evaluation of the sealer-dentine interface of three sealers. Iran Endod J.

[8] McMichen FR, Pearson G, Rahbaran S, Gulabivala K (2003). A comparative study of selected physical properties of five root-canal sealers. Int Endod J.

[9] Schäfer E, Bering N, Bürklein S (2015). Selected physicochemical properties of AH Plus, EndoREZ and RealSeal SE root canal sealers. Odontology.

[10] Baras BH, Melo MA, Thumbigere-Math V (2020). Novel bioactive and therapeutic root canal sealers with antibacterial and remineralization properties. Materials (Basel).

[11] Steier L, de Figueiredo JAP, Belli S (2010). Comparison of the interface dentin-endodontic sealer using two SEM magnifications. Rev Odonto Ciênc.

[12] Hakke Patil A, Patil AG, Shaikh S, Bhandarkar S, Moharir A, Sharma A (2024). Comparative evaluation of the sealing ability of mineral trioxide aggregate (MTA)-based, resin-based, and zinc oxide eugenol root canal sealers: an in vitro study. Cureus.

[13] Ørstavik D, Nordahl I, Tibballs JE (2001). Dimensional change following setting of root canal sealer materials. Dent Mater.

[14] Kim YK, Mai S, Haycock JR, Kim SK, Loushine RJ, Pashley DH, Tay FR (2009). The self-etching potential of RealSeal versus RealSeal SE. J Endod.

[15] Babb BR, Loushine RJ, Bryan TE (2009). Bonding of self-adhesive (self-etching) root canal sealers to radicular dentin. J Endod.

[16] Al-Haddad A, Che Ab Aziz ZA (2016). Bioceramic-based root canal sealers: a review. Int J Biomater.

[17] Nagar N, Kumar N (2018). A comparative clinical evaluation of a bioceramic root canal sealer with MTA-based sealer, resin-based sealer, and zinc oxide-based sealer - an in vivo study. IOSR J Dent Med Sci.

[18] Hollanda AC, Estrela CR, Decurcio Dde A, Silva JA, Estrela C (2009). Sealing ability of three commercial resin-based endodontic sealers. Gen Dent.

[19] Pommel L, About I, Pashley D, Camps J (2003). Apical leakage of four endodontic sealers. J Endod.

[20] De Almeida WA, Leonardo MR, Tanomaru Filho M, Silva LA (2000). Evaluation of apical sealing of three endodontic sealers. Int Endod J.

